# The Promise, Pitfalls, and Practicalities of Precision Education in Obstetrics and Gynecology

**DOI:** 10.1097/og9.0000000000000140

**Published:** 2026-01-08

**Authors:** Laura Baecher-Lind, Alyssa Stephenson-Famy, Katherine T. Chen, Angela Fleming, Christine Kim, Silka Patel, Jonathan Schaffir, Shireen Madani Sims, Tammy Sonn, Jill M. Sutton, Hedwige Saint Louis

**Affiliations:** Tufts School of Medicine, Boston, Massachusetts; Department of Obstetrics and Gynecology, University of Washington, Seattle, Washington; Icahn School of Medicine at Mount Sinai, New York, New York; Department of Obstetrics and Gynecology, Corewell Health, Farmington Hills, Michigan; Department of Obstetrics and Gynecology at the University of California, Irvine, California; Department of Gynecology and Obstetrics, Johns Hopkins School of Medicine, Baltimore, Maryland; Department of Obstetrics and Gynecology, Wexner Medical Center, The Ohio State University, Columbus, Ohio; Department of Obstetrics and Gynecology, University of Florida College of Medicine, Gainesville, Florida; Department of Obstetrics and Gynecology, Washington University School of Medicine, St. Louis, Missouri; Department of Obstetrics and Gynecology, ECU Brody School of Medicine, ECU Health Medical Center, Greenville, North Carolina; and Department of Obstetrics and Gynecology, Morehouse School of Medicine, Atlanta, Georgia.

## Abstract

Precision education is a relatively new concept in medical education that incorporates elements of community-centered practice, continuous quality improvement, and competency-based medical education and is fueled by frequent, reliable, unbiased data on learner performance. Precision education has been described as “the next era of medical education.” However, the concept of precision education remains unfamiliar to many physicians, and the cost and expertise associated with precision education pose a substantial barrier to its adoption. This review aims to support educators and leaders in obstetrics and gynecology to better understand precision education and to provide practical, cost-effective precision education strategies that can be implemented into obstetrics and gynecology clinical education programs.

## Abstract 2

Precision education offers potential to advance medical education and health care outcomes in obstetrics and gynecology and can be adopted incrementally and practically across settings.

Precision education has been described as “the next era of medical education” that holds the promise to “improve the health of the nation” by the American Medical Association.^1–3^ Funding opportunities and programming have been earmarked to help advance precision education.^3^ However, the concept of precision education remains unfamiliar to many clinical educators and leaders. In addition, the cost associated with precision education limits the ability of many medical schools and clinical departments to incorporate these practices into their educational programs.^1^ This review aims to support educators and leaders in obstetrics and gynecology to better understand the concepts and obstacles of precision education and to provide practical, cost-effective strategies that can be implemented as part of a step-wise approach toward adopting precision education within educational programs.

## WHAT IS PRECISION EDUCATION?

Although the term precision education is relatively novel, the concepts on which precision education is built are not. Precision education weaves together concepts of competency-based medical education, quality improvement, community-based care, and growth mindset.^4–7^ Just as precision medicine indicates a medical approach that considers each patient's unique genetics, physiology, history, and health care goals, precision education indicates an educational approach that considers each student's unique skills, capabilities, experiences, and educational goals. Rather than an identical educational program that each learner completes in lockstep with all other learners, a precision education approach is inherently flexible and individually tailored. A learner who accomplishes a learning outcome quickly would move on to other experiences; that same learner might require additional time to master other learning outcomes and would receive additional support and time in those domains. Similarly, learning outcomes are guided by the health care needs of the patients and communities whom organizations serve and therefore may fluctuate over time and vary between organizations.^3,7^

**Fig. 1.**
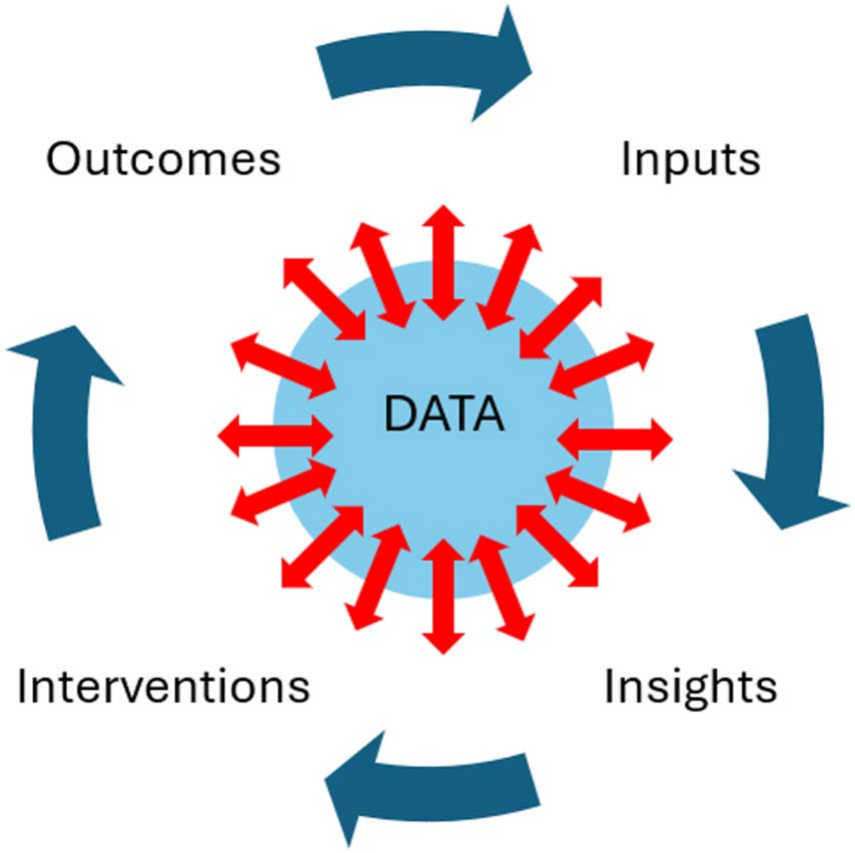
Conceptual model of precision education (PE). Central to PE is reliable, unbiased, and frequent data that transmit to and from learners as they interface with patients, educators, and technologies. Educational and health outcomes associated with a learner are gathered and become the inputs. Analysis of the input data generates insights into learner performance. Opportunities for growth are generated as interventions for the learner to undertake. This may include reading materials, simulation, or clinical activities. The results achieved by the learner from those interventions are used to further inform the system as new, updated inputs.

Precision education intentionally adopts a plan–do–study–act style approach familiar to quality improvement initiatives, with a strong emphasis on frequent, immediate, and specific feedback to learners (Fig. 1). Traditionally, educators struggle to provide the robust, high-value feedback that students expect.^8^ Precision education relies on largely automated systems that provide specific, immediate, and high-value feedback to students as they work and learn in the clinical setting.^1,9^ This feedback is independent of the supervising clinician and may involve tools supported by artificial intelligence (AI). Examples of AI-guided feedback include an automated review of a student's clinical note with a suggested additional diagnosis to consider, an automated summary of conditions logged by a learner with links to additional reading materials specific to those conditions, real-time pressure feedback using pressure-sensing gloves to determine the accuracy of breast or pelvic examinations, or an automated report on the proportion of time a student spent looking at the patient during clinical encounters. These feedback systems can also be used to help determine when a student has achieved certain learning outcomes and can therefore be directed to other educational experiences.^9^

*Baecher-Lind. Precision Education in Obstetrics and Gynecology. Obstet Gynecol 2025*.

Because precision education relies heavily on automated and standardized feedback to students, bias related to clinical performance can be mitigated.^10^ Systems that rely on large language models should be designed to reduce rather than amplify biases.^11^ Technology-based systems that do not rely on large language models such as haptics-informed mannequins, motion-capture, and ambient listening technologies can allow accurate and unbiased assessment of students' performance. Collectively, these technology-driven assessments can be used to inform student assessments and promotion readiness.^12^

Precision education requires learners to be engaged with and responsible for their own educational needs. Learners who delay incorporating feedback will take longer to achieve learning goals, whereas learners who are receptive to feedback and implement strategies to address them will succeed more rapidly and to a greater degree. Learners who are educated and trained with a precision education approach are therefore expected to be more facile with the lifelong learning required of physicians to meet the evolving health care needs of the future.^13^

Lastly, precision education is committed to keeping patients, communities, and health care outcomes at the center of medical education.^1,7^ Precision education recognizes that the goal of medical education writ large is to meet the health care needs of patients and communities, that our mandate as medical educators is to develop a workforce that fulfills our societal commitment to optimize health and life for our populace. In that sense, precision education embraces patient and community partnerships and public health needs and outcomes in determining the learning objectives that drive medical education.^7^

## PRECISION EDUCATION IN OBSTETRICS AND GYNECOLOGY

Most medical schools require a 6-week obstetrics and gynecology clerkship during the core clerkship year.^14^ Students on the obstetrics and gynecology clerkship are typically assigned a schedule at the start of the clerkship and deemed to have successfully completed the clerkship once the time frame passes regardless of actual student experiences. Clerkship faculty may or may not encourage students to read about the patients and conditions they saw to supplement their clinical learning. Learning objectives are generated by clerkship leadership and align with institutional objectives and national specialty guidelines.^15^ Students may or may not receive feedback from preceptors in addition to one or two meetings with clerkship leadership. Assessment often consists of a clinical performance evaluation, along with other measures such as standardized examination score or Objective Structured Clinical Examination score established by the medical school.

**Fig. 2.**
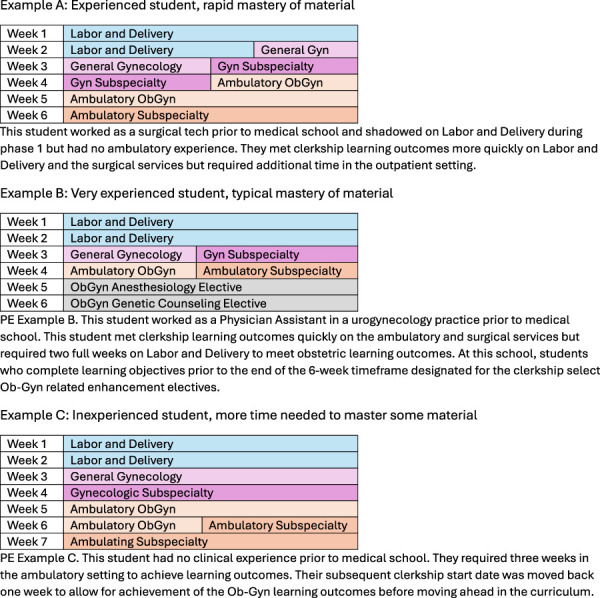
Examples of competency-based, precision education (PE) approaches to the obstetrics and gynecology (ObGyn) clerkship.

An obstetrics and gynecology clerkship that embraces precision education principles may start by involving patients or community members in the development of learning objectives for the clerkship experience to account for the health care needs and goals of the community. Students would begin the clerkship with a general schedule that is malleable depending on students' experiences, aptitudes, and rate of learning (Fig. 2). Assessment of student performance would occur throughout the day with automated assessments such as AI-driven evaluation of students' documentation or haptic-informed feedback about students' physical examinations. Feedback would be automatically paired with suggestions for improvement in addition to AI-driven suggestions for reading assignments. Patient logs could be completed not on the basis of student recollection of their experiences but instead by automation with electronic medical records linking to educational learning platforms, creating a dashboard that can be used by students and educators to gauge progress. Prompts would be driven by this automation to let learners and educators know when they have achieved learning objectives and can move on to additional experiences.

*Baecher-Lind. Precision Education in Obstetrics and Gynecology. Obstet Gynecol 2025*.

## BARRIERS TO IMPLEMENTING PRECISION EDUCATION

There are several notable barriers to implementing precision education (Box 1). The infrastructure and talent investment needed to set up AI-generated feedback systems, to integrate electronic medical records with educational platforms, and to purchase technologies for haptic or attention-tracking devices is cost prohibitive for many organizations.^1^ Most medical schools and obstetrics and gynecology clinical departments are not currently resourced to deliver on the promise of precision education. Calls to increase funding at the federal and state levels to support precision education given the population health and equity benefits have so far been unmet.^1,2^

Box 1. **Barriers to Widespread Implementation of Precision Education in Medical Education**

Cost of expertise to develop and manage precision education systems

Cost of integration between health care/EHR and educational learning platforms*

Cost of technologies necessary for reliable, unbiased, frequent feedback

Distributed medical education models/many clinical affiliates

Logistic challenges with cyclical, fixed-date events including orientations and residency application activities

Logistic challenges with variation in start and end times between clerkships

Logistical challenges with variation within a clerkship of student volume per service

National and state licensing requirements that are time based†

Policies that prohibit sharing of student information across disciplines

EHR, electronic health record.

*Requires an organization-specific large language model approach to protect both patient and student data.

†Requires legislation to allow a competency-based approach to meet licensing and other practice requirements for physicians.

Smaller medical schools or departments with a single clinical site will be more readily able to implement precision education compared with programs that rely on many affiliates in a distributed clinical model. Precision education relies on the integration of electronic medical records with educational platforms and programming; distributed schools may send students to dozens of clinical sites, each with their own systems and electronic medical records, thereby functionally precluding widespread use of precision education across sites.^16^

Regardless of the number of clinical affiliates, all medical schools will have to contend with the variation of students completing clinical requirements at different paces. Students demonstrating competency in a short period of time may need to schedule shelf examinations or clinical assessments sooner than those requiring longer clerkship durations. Short notice for students embarking on electives or moving onto other clerkships, including institutional onboarding for those sites, will need to be managed. Cyclical events such as clerkship orientations, transition to residency courses, commencement, and others may need to be delivered differently.

Medical schools vary in their approach to sharing student assessment information across disciplines, with some schools prohibiting the sharing of prior student performance to clerkship directors in order to allow the student the opportunity to start fresh in each clerkship (the “clean slate” approach), whereas other schools intentionally share student performance information as students embark on a clerkship to allow longitudinal growth and development (the “feed forward” approach). In precision education approaches, student performance data are often proactively shared with the student, course and clerkship directors, and coaches. Schools with a clean slate approach may need to consider strategies to allow student performance data to be shared across disciplines to allow longitudinal and tailored learning experiences.

National and state licensing requirements are rooted in time-based rather than competency-based frameworks.^17^ A flexible, competency-based approach would potentially prevent graduates from meeting license requirements should they have fulfilled learning objectives more rapidly than current systems require. Full implementation of precision education may require changes at the national and state levels regarding accreditation and licensing requirements for physicians, a process that will likely be slow and fragmented.

The community-based determination of learning outcomes assumes that learners will be practicing in that community; however, there is considerable dispersion of students after graduation from medical school. Delivering on societal goals may be difficult for organizations with workforces trained using learning objectives specific to a different population. Patient and population health outcomes must be monitored to determine whether the precision education approach truly moves the needle on pervasive health care shortcomings and inequities inherent to the U.S. health care system. Although data support the efficacy of precision education in educational settings,^18^ data specific to the influence of precision education on health care outcomes in medical education are promising but sparse.^7,19^

## STRATEGIES TO INCORPORATE ELEMENTS OF PRECISION EDUCATION INTO TRADITIONAL OBSTERICS AND GYNECOLOGY MEDICAL EDUCATION

There are few well-developed, fully realized programs of precision education in U.S. medical schools today.^20^ The barriers may seem daunting and dissuade educators from experimenting with the principles of precision education in their programs. Given the limited capability for wholesale precision education at this time, the adoption of precision education will likely occur in a more cost-effective, piecemeal, and gradual manner.

Educators and leaders in obstetrics and gynecology can incorporate aspects of precision education into obstetrics and gynecology learning environments without an institution-wide transition to this approach. Initiatives or technologies that enhance precision education for clerkship students can be scaled to also benefit other learners such as residents or fellows and can even be used to identify practice patterns and improvement insights for faculty. For example, AI-guided review of prescribing patterns has been effective in training students on responsible prescribing, guiding residents on cost considerations of their prescription choices, and giving insight to faculty on how their prescribing practices differ from the practices of their peers.^21^ Obstetrics and gynecology may be uniquely positioned among specialties to incorporate precision education principles into educational programs, given the many clinical settings and the unique combination of procedural and ambulatory elements that are involved that allow leverage and collaboration across specialties and departments. Table 1 provides a list of practical strategies to incorporate precision education principles into obstetrics and gynecology learning environments.

**Table 1.** Practical Precision Education Strategies That Can Enhance Traditional Obstetrics and Gynecology Medical Education

Strategy	Example(s)	Pathway to Implementation
AI-driven analysis of EHR documentation to allow EHR meta-data	Learners receive AI-generated feedback after documenting an obstetrics triage patient encounter in the EHR, including recommendations for a more concise note and an additional diagnosis to consider. Learners receive a summary of the diagnoses they entered that day in the ambulatory setting, along with suggested supplemental readings to complement their clinical learning.	Collaborate with technology services responsible for EHR within the clinical enterprise and learning platforms within the medical school to integrate systems and to develop a small or moderate language model specific to the organization that is HIPAA and FERPA compliant. Collaborate with technical services to develop a program that suggests readings based on learners' clinical experiences as captured in the EHR. For example, a student who reviewed the chart of a patient with a diagnosis of preeclampsia receives a list of suggested readings and national guidelines related to preeclampsia in their educational platform at the end of the day.
Haptics-informed procedural skills assessment	Learners participate in a breast examination simulation session during clerkship orientation and again in a summative assessment at clerkship completion that uses haptics-equipped breast models that provide feedback regarding the pressure used during examinations.	Consider cost sharing with GME for purchases of haptics expected to have a higher educational return on investment for a particular obstetrics and gynecology department, which may include examination mannequins or procedural trainers.
Ambient listening technology	Sensors in examination rooms monitor and evaluate clinical encounters; learners receive synopsis of their interpersonal and communication skills, including strategies to improve efficiency, accuracy, and patient experience.	Consider cost sharing with GME and the clinical enterprise for purchases of ambient listening devices that integrate with EHR because these technologies have applications and benefits for both educational and clinical practice (productivity, patient experience, faculty retention).
Motion-tracking/motion-capture technology	Sensors in examination rooms detect where clinicians' attention is focused throughout a patient encounter and calculate the proportion of time spent looking at patients during history taking and counseling.	Consider cost haring with GME and the clinical enterprise for purchases of motion-capture technologies because these technologies have applications and benefits for both educational and clinical practice (productivity and patient experience).
Patient and community engagement in setting learning outcomes	Educators include patients and community members on Curriculum Committees, residency Clinical Competency Committees, or other working groups when learning outcomes are established.	Consider piloting the inclusion of a patient or a community member as a standing member on an education-focused departmental committee or as a guest when learning outcomes are set; consider surveying community members on their goals for the training of their community's health care professionals through survey, focus group, or meeting with liaisons.
Clerkship scheduling that is flexible and modifiable by achievement of learning outcomes or student goals	Students meeting required obstetrics and gynecology clinical experiences and achieving learning outcomes in time frames shorter than the allotted time for the clerkship can then select an intraclerkship elective. Students starting the obstetrics and gynecology clerkship demonstrating competency in breast and pelvic examination skills from a preclerkship course and the Family Medicine clerkship can be scheduled for fewer ambulatory sessions and offered a self-directed learning experience instead.	Collaborate with medical school leadership to further develop an approach that meets school requirements with obstetrics and gynecology educational leaders to ensure valid assessments of when educational competencies are met and with students to develop electives and self-directed educational experiences.

AI, artificial intelligence; EHR, electronic health record; HIPAA, Health Insurance Portability and Accountability Act; FERPA, Family Educational Rights and Privacy Act; GME, graduate medical education.

## CONCLUSION

Precision education offers great potential to advance accuracy and equity within medical education, to improve efficiency of physician workforce training, and to better align the physician workforce with the communities we serve. Significant barriers impede our path from current state to this more equitable, reliable, and patient-centered future. Rather than letting these foreboding barriers intimidate us, leading us to potentially avoid or even reject the promise of precision education, we can work creatively to incrementally adopt precision education into our clinical and educational spaces. In this way, students, residents, and even faculty can benefit from the rapid advancements in technology largely powered by AI and the use of machines and technologies to enhance learning and practice insights and to more effectively realize our mission of optimizing the health and well-being of patients now and in the future.
